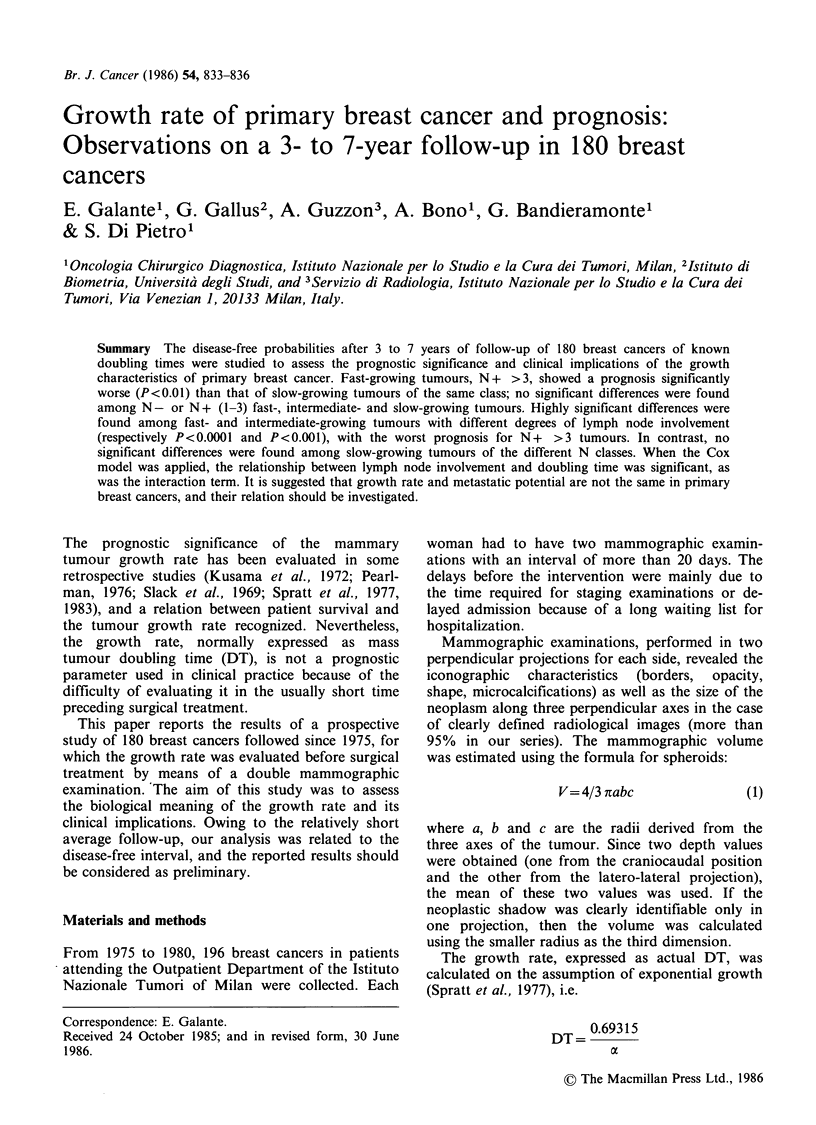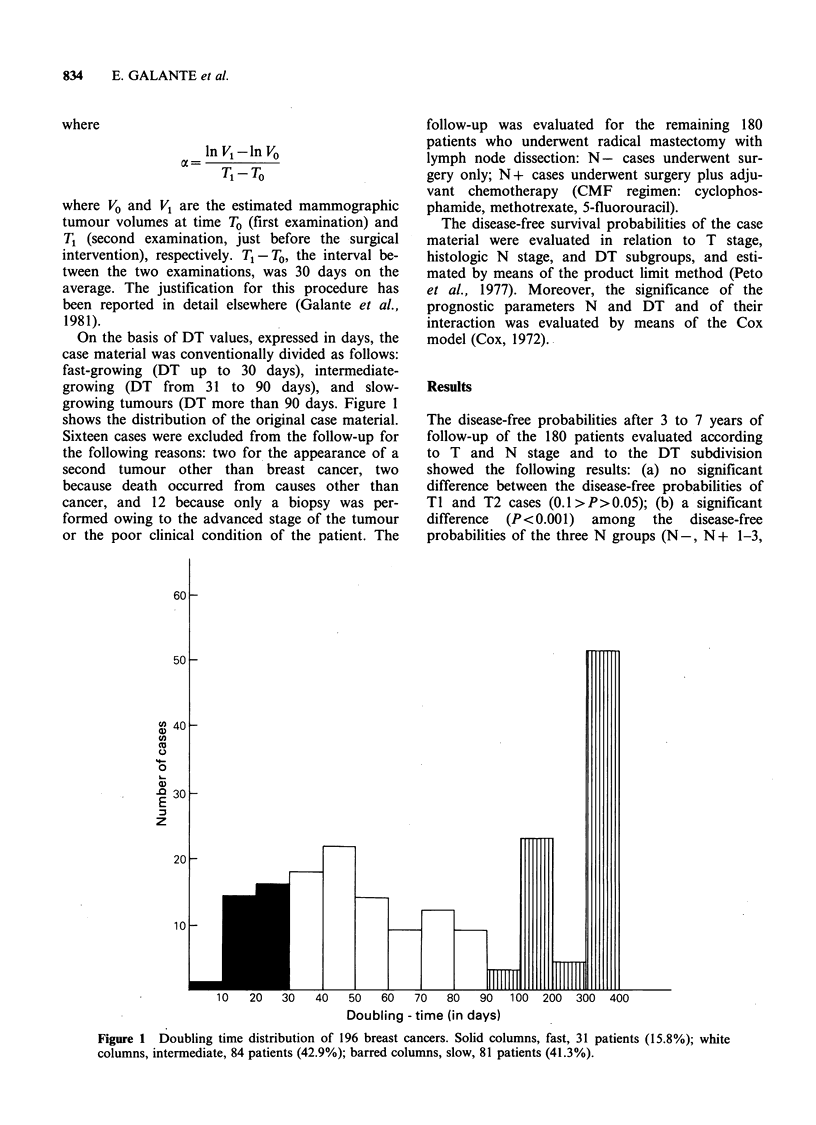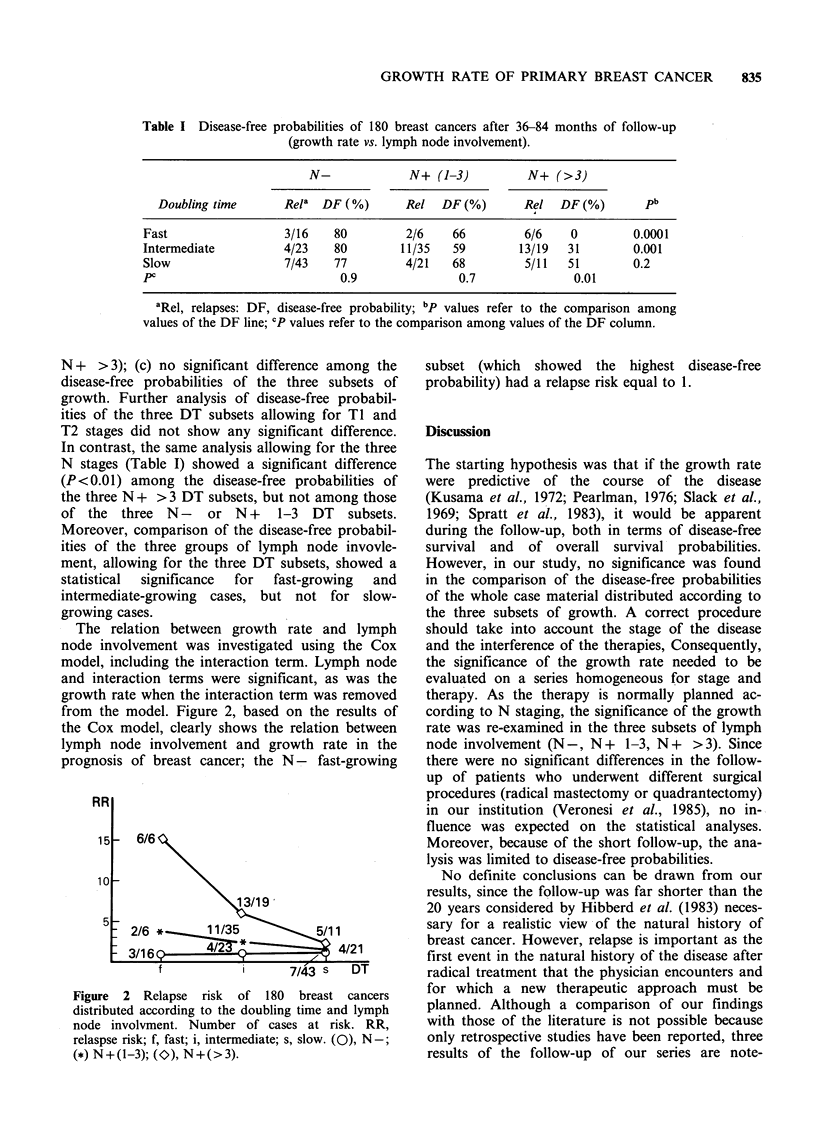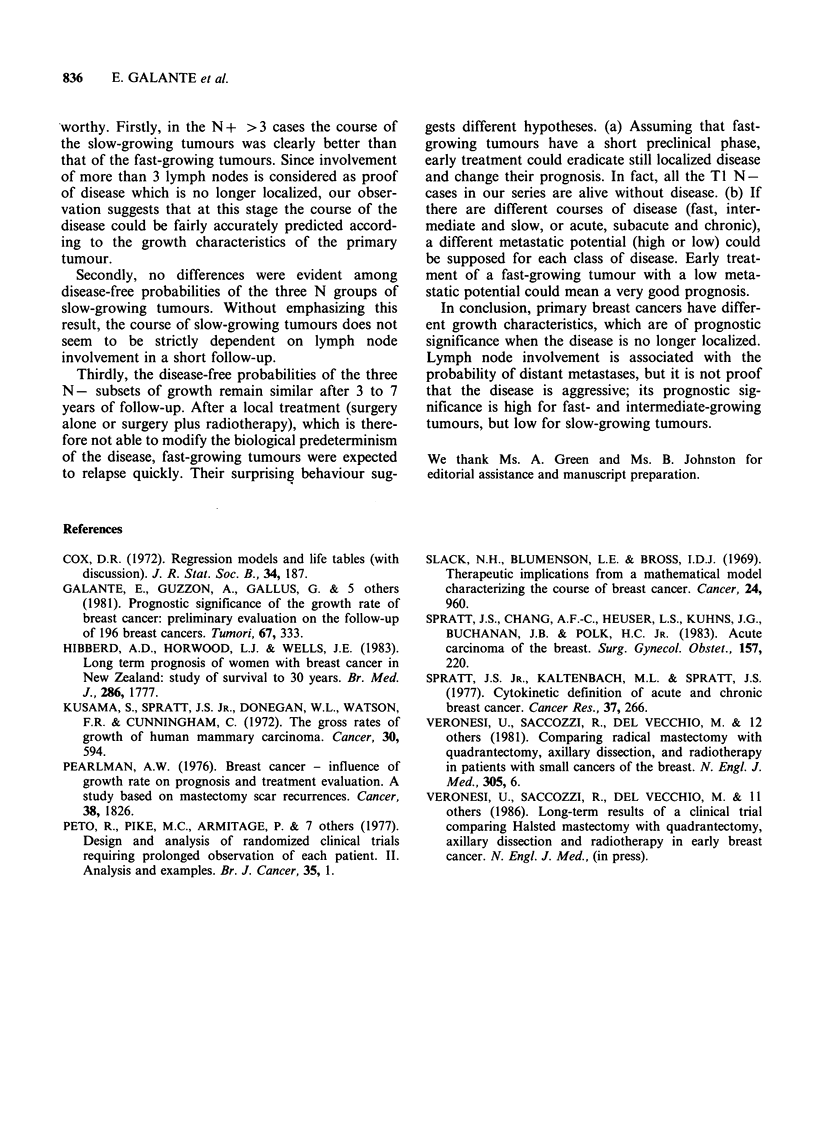# Growth rate of primary breast cancer and prognosis: observations on a 3- to 7-year follow-up in 180 breast cancers.

**DOI:** 10.1038/bjc.1986.247

**Published:** 1986-11

**Authors:** E. Galante, G. Gallus, A. Guzzon, A. Bono, G. Bandieramonte, S. Di Pietro

## Abstract

The disease-free probabilities after 3 to 7 years of follow-up of 180 breast cancers of known doubling times were studied to assess the prognostic significance and clinical implications of the growth characteristics of primary breast cancer. Fast-growing tumours, N+ greater than 3, showed a prognosis significantly worse (P less than 0.01) than that of slow-growing tumours of the same class; no significant differences were found among N- or N+ (1-3) fast-, intermediate- and slow-growing tumours. Highly significant differences were found among fast- and intermediate-growing tumours with different degrees of lymph node involvement (respectively P less than 0.0001 and P less than 0.001), with the worst prognosis for N+ greater than 3 tumours. In contrast, no significant differences were found among slow-growing tumours of the different N classes. When the Cox model was applied, the relationship between lymph node involvement and doubling time was significant, as was the interaction term. It is suggested that growth rate and metastatic potential are not the same in primary breast cancers, and their relation should be investigated.


*Br. J. Cancer* (1986) **54**, 833–836

### Growth rate of primary breast cancer and prognosis: Observations on a 3- to 7-year follow-up in 180 breast cancers

E. Galante[1], G. Gallus[2], A. Guzzon[3], A. Bono[1], G. Bandieramonte[1] & S. Di Pietro[1]

[1]*Oncologia Chirurgico Diagnostica, Istituto Nazionale per lo Studio e la Cura dei Tumori, Milan,* [2]*Istituto di Biometria, Università degli Studi, and* [3]*Servizio di Radiologia, Istituto Nazionale per lo Studio e la Cura dei Tumori, Via Venezian 1, 20133 Milan, Italy.*

**Summary** The disease-free probabilities after 3 to 7 years of follow-up of 180 breast cancers of known doubling times were studied to assess the prognostic significance and clinical implications of the growth characteristics of primary breast cancer. Fast-growing tumours, N+ >3, showed a prognosis significantly worse ($P<0.01$) than that of slow-growing tumours of the same class; no significant differences were found among N− or N+ (1–3) fast-, intermediate- and slow-growing tumours. Highly significant differences were found among fast- and intermediate-growing tumours with different degrees of lymph node involvement (respectively $P<0.0001$ and $P<0.001$), with the worst prognosis for N+ >3 tumours. In contrast, no significant differences were found among slow-growing tumours of the different N classes. When the Cox model was applied, the relationship between lymph node involvement and doubling time was significant, as was the interaction term. It is suggested that growth rate and metastatic potential are not the same in primary breast cancers, and their relation should be investigated.

The prognostic significance of the mammary tumour growth rate has been evaluated in some retrospective studies (Kusama *et al.*, 1972; Pearlman, 1976; Slack *et al.*, 1969; Spratt *et al.*, 1977, 1983), and a relation between patient survival and the tumour growth rate recognized. Nevertheless, the growth rate, normally expressed as mass tumour doubling time (DT), is not a prognostic parameter used in clinical practice because of the difficulty of evaluating it in the usually short time preceding surgical treatment.

This paper reports the results of a prospective study of 180 breast cancers followed since 1975, for which the growth rate was evaluated before surgical treatment by means of a double mammographic examination. The aim of this study was to assess the biological meaning of the growth rate and its clinical implications. Owing to the relatively short average follow-up, our analysis was related to the disease-free interval, and the reported results should be considered as preliminary.

### Materials and methods

From 1975 to 1980, 196 breast cancers in patients attending the Outpatient Department of the Istituto Nazionale Tumori of Milan were collected. Each woman had to have two mammographic examinations with an interval of more than 20 days. The delays before the intervention were mainly due to the time required for staging examinations or delayed admission because of a long waiting list for hospitalization.

Mammographic examinations, performed in two perpendicular projections for each side, revealed the iconographic characteristics (borders, opacity, shape, microcalcifications) as well as the size of the neoplasm along three perpendicular axes in the case of clearly defined radiological images (more than 95% in our series). The mammographic volume was estimated using the formula for spheroids:

$$V=4/3\,\pi abc \qquad (1)$$

where $a$, $b$ and $c$ are the radii derived from the three axes of the tumour. Since two depth values were obtained (one from the craniocaudal position and the other from the latero-lateral projection), the mean of these two values was used. If the neoplastic shadow was clearly identifiable only in one projection, then the volume was calculated using the smaller radius as the third dimension.

The growth rate, expressed as actual DT, was calculated on the assumption of exponential growth (Spratt *et al.*, 1977), i.e.

$$\mathrm{DT}=\frac{0.69315}{\alpha}$$

Correspondence: E. Galante.
Received 24 October 1985; and in revised form, 30 June 1986.

© The Macmillan Press Ltd., 1986

where

$$\alpha = \frac{\ln V_1 - \ln V_0}{T_1 - T_0}$$

where $V_0$ and $V_1$ are the estimated mammographic tumour volumes at time $T_0$ (first examination) and $T_1$ (second examination, just before the surgical intervention), respectively. $T_1 - T_0$, the interval between the two examinations, was 30 days on the average. The justification for this procedure has been reported in detail elsewhere (Galante *et al.*, 1981).

On the basis of DT values, expressed in days, the case material was conventionally divided as follows: fast-growing (DT up to 30 days), intermediate-growing (DT from 31 to 90 days), and slow-growing tumours (DT more than 90 days. Figure 1 shows the distribution of the original case material. Sixteen cases were excluded from the follow-up for the following reasons: two for the appearance of a second tumour other than breast cancer, two because death occurred from causes other than cancer, and 12 because only a biopsy was performed owing to the advanced stage of the tumour or the poor clinical condition of the patient. The follow-up was evaluated for the remaining 180 patients who underwent radical mastectomy with lymph node dissection: N− cases underwent surgery only; N+ cases underwent surgery plus adjuvant chemotherapy (CMF regimen: cyclophosphamide, methotrexate, 5-fluorouracil).

The disease-free survival probabilities of the case material were evaluated in relation to T stage, histologic N stage, and DT subgroups, and estimated by means of the product limit method (Peto *et al.*, 1977). Moreover, the significance of the prognostic parameters N and DT and of their interaction was evaluated by means of the Cox model (Cox, 1972).

### Results

The disease-free probabilities after 3 to 7 years of follow-up of the 180 patients evaluated according to T and N stage and to the DT subdivision showed the following results: (a) no significant difference between the disease-free probabilities of T1 and T2 cases ($0.1 > P > 0.05$); (b) a significant difference ($P < 0.001$) among the disease-free probabilities of the three N groups (N−, N+ 1–3,

**Figure 1** Doubling time distribution of 196 breast cancers. Solid columns, fast, 31 patients (15.8%); white columns, intermediate, 84 patients (42.9%); barred columns, slow, 81 patients (41.3%).

**Table I** Disease-free probabilities of 180 breast cancers after 36–84 months of follow-up (growth rate *vs*. lymph node involvement).

| | *N−* | | *N+ (1-3)* | | *N+ (>3)* | | |
|---|---|---|---|---|---|---|---|
| *Doubling time* | *Rel*[a] | *DF* (%) | *Rel* | *DF* (%) | *Rel* | *DF* (%) | *P*[b] |
| Fast | 3/16 | 80 | 2/6 | 66 | 6/6 | 0 | 0.0001 |
| Intermediate | 4/23 | 80 | 11/35 | 59 | 13/19 | 31 | 0.001 |
| Slow | 7/43 | 77 | 4/21 | 68 | 5/11 | 51 | 0.2 |
| *P*[c] | | 0.9 | | 0.7 | | 0.01 | |

[a]Rel, relapses: DF, disease-free probability; [b]*P* values refer to the comparison among values of the DF line; [c]*P* values refer to the comparison among values of the DF column.

N+ >3); (c) no significant difference among the disease-free probabilities of the three subsets of growth. Further analysis of disease-free probabilities of the three DT subsets allowing for T1 and T2 stages did not show any significant difference. In contrast, the same analysis allowing for the three N stages (Table I) showed a significant difference ($P<0.01$) among the disease-free probabilities of the three N+ >3 DT subsets, but not among those of the three N− or N+ 1–3 DT subsets. Moreover, comparison of the disease-free probabilities of the three groups of lymph node invovlement, allowing for the three DT subsets, showed a statistical significance for fast-growing and intermediate-growing cases, but not for slow-growing cases.

The relation between growth rate and lymph node involvement was investigated using the Cox model, including the interaction term. Lymph node and interaction terms were significant, as was the growth rate when the interaction term was removed from the model. Figure 2, based on the results of the Cox model, clearly shows the relation between lymph node involvement and growth rate in the prognosis of breast cancer; the N− fast-growing subset (which showed the highest disease-free probability) had a relapse risk equal to 1.

**Figure 2** Relapse risk of 180 breast cancers distributed according to the doubling time and lymph node involvment. Number of cases at risk. RR, relaspse risk; f, fast; i, intermediate; s, slow. (○), N−; (∗) N+(1–3); (◇), N+(>3).

### Discussion

The starting hypothesis was that if the growth rate were predictive of the course of the disease (Kusama *et al.*, 1972; Pearlman, 1976; Slack *et al.*, 1969; Spratt *et al.*, 1983), it would be apparent during the follow-up, both in terms of disease-free survival and of overall survival probabilities. However, in our study, no significance was found in the comparison of the disease-free probabilities of the whole case material distributed according to the three subsets of growth. A correct procedure should take into account the stage of the disease and the interference of the therapies, Consequently, the significance of the growth rate needed to be evaluated on a series homogeneous for stage and therapy. As the therapy is normally planned according to N staging, the significance of the growth rate was re-examined in the three subsets of lymph node involvement (N−, N+ 1–3, N+ >3). Since there were no significant differences in the follow-up of patients who underwent different surgical procedures (radical mastectomy or quadrantectomy) in our institution (Veronesi *et al.*, 1985), no influence was expected on the statistical analyses. Moreover, because of the short follow-up, the analysis was limited to disease-free probabilities.

No definite conclusions can be drawn from our results, since the follow-up was far shorter than the 20 years considered by Hibberd *et al.* (1983) necessary for a realistic view of the natural history of breast cancer. However, relapse is important as the first event in the natural history of the disease after radical treatment that the physician encounters and for which a new therapeutic approach must be planned. Although a comparison of our findings with those of the literature is not possible because only retrospective studies have been reported, three results of the follow-up of our series are noteworthy. Firstly, in the N+ >3 cases the course of the slow-growing tumours was clearly better than that of the fast-growing tumours. Since involvement of more than 3 lymph nodes is considered as proof of disease which is no longer localized, our observation suggests that at this stage the course of the disease could be fairly accurately predicted according to the growth characteristics of the primary tumour.

Secondly, no differences were evident among disease-free probabilities of the three N groups of slow-growing tumours. Without emphasizing this result, the course of slow-growing tumours does not seem to be strictly dependent on lymph node involvement in a short follow-up.

Thirdly, the disease-free probabilities of the three N− subsets of growth remain similar after 3 to 7 years of follow-up. After a local treatment (surgery alone or surgery plus radiotherapy), which is therefore not able to modify the biological predeterminism of the disease, fast-growing tumours were expected to relapse quickly. Their surprising behaviour suggests different hypotheses. (a) Assuming that fast-growing tumours have a short preclinical phase, early treatment could eradicate still localized disease and change their prognosis. In fact, all the T1 N− cases in our series are alive without disease. (b) If there are different courses of disease (fast, intermediate and slow, or acute, subacute and chronic), a different metastatic potential (high or low) could be supposed for each class of disease. Early treatment of a fast-growing tumour with a low metastatic potential could mean a very good prognosis.

In conclusion, primary breast cancers have different growth characteristics, which are of prognostic significance when the disease is no longer localized. Lymph node involvement is associated with the probability of distant metastases, but it is not proof that the disease is aggressive; its prognostic significance is high for fast- and intermediate-growing tumours, but low for slow-growing tumours.

We thank Ms. A. Green and Ms. B. Johnston for editorial assistance and manuscript preparation.

### References

COX, D.R. (1972). Regression models and life tables (with discussion). *J. R. Stat. Soc. B.*, **34**, 187.

GALANTE, E., GUZZON, A., GALLUS, G. & 5 others (1981). Prognostic significance of the growth rate of breast cancer: preliminary evaluation on the follow-up of 196 breast cancers. *Tumori*, **67**, 333.

HIBBERD, A.D., HORWOOD, L.J. & WELLS, J.E. (1983). Long term prognosis of women with breast cancer in New Zealand: study of survival to 30 years. *Br. Med. J.*, **286**, 1777.

KUSAMA, S., SPRATT, J.S. JR., DONEGAN, W.L., WATSON, F.R. & CUNNINGHAM, C. (1972). The gross rates of growth of human mammary carcinoma. *Cancer*, **30**, 594.

PEARLMAN, A.W. (1976). Breast cancer – influence of growth rate on prognosis and treatment evaluation. A study based on mastectomy scar recurrences. *Cancer*, **38**, 1826.

PETO, R., PIKE, M.C., ARMITAGE, P. & 7 others (1977). Design and analysis of randomized clinical trials requiring prolonged observation of each patient. II. Analysis and examples. *Br. J. Cancer*, **35**, 1.

SLACK, N.H., BLUMENSON, L.E. & BROSS, I.D.J. (1969). Therapeutic implications from a mathematical model characterizing the course of breast cancer. *Cancer*, **24**, 960.

SPRATT, J.S., CHANG, A.F.-C., HEUSER, L.S., KUHNS, J.G., BUCHANAN, J.B. & POLK, H.C. JR. (1983). Acute carcinoma of the breast. *Surg. Gynecol. Obstet.*, **157**, 220.

SPRATT, J.S. JR., KALTENBACH, M.L. & SPRATT, J.S. (1977). Cytokinetic definition of acute and chronic breast cancer. *Cancer Res.*, **37**, 266.

VERONESI, U., SACCOZZI, R., DEL VECCHIO, M. & 12 others (1981). Comparing radical mastectomy with quadrantectomy, axillary dissection, and radiotherapy in patients with small cancers of the breast. *N. Engl. J. Med.*, **305**, 6.

VERONESI, U., SACCOZZI, R., DEL VECCHIO, M. & 11 others (1986). Long-term results of a clinical trial comparing Halsted mastectomy with quadrantectomy, axillary dissection and radiotherapy in early breast cancer. *N. Engl. J. Med.*, (in press).